# The Praise and Price of Pokémon GO: A Qualitative Study of Children’s and Parents’ Experiences

**DOI:** 10.2196/games.8979

**Published:** 2018-01-03

**Authors:** Anna-Karin Lindqvist, Darla Castelli, Josef Hallberg, Stina Rutberg

**Affiliations:** ^1^ Health and Rehabilitation Department of Health Sciences Luleå University of Technology Luleå Sweden; ^2^ Kinetic Kidz Lab Department of Kinesiology and Health Education University of Texas at Austin Austin, TX United States; ^3^ Department of Computer Science Electrical and Space Engineering Luleå University of Technology Luleå Sweden

**Keywords:** child, cell phone, parents, exercise, mobile apps

## Abstract

**Background:**

Physical activity has multiple health benefits; however, the majority of children around the world do not attain the recommended levels of daily physical activity. Research has shown that the game *Poké* *mon GO* has increased the amount of physical activity of players and that the game has the potential to reach populations that traditionally have low levels of physical activity. Therefore, there is a need to understand which game components can promote initial and sustained physical activity. By using a qualitative research approach, it is possible to achieve rich descriptions and enhance a deep understanding of the components promoting physical activity among children in a game such as *Poké* *mon GO.*

**Objective:**

The objective of this study was to explore children’s and parents’ experiences playing *Poké* *mon GO*.

**Methods:**

Eight families comprising 13 children (aged 7-12 years) and 9 parents were selected using purposeful sampling. Data collected using focus groups were analyzed using qualitative latent content analysis.

**Results:**

The following three themes were revealed: (1) exciting and enjoyable exploration; (2) dangers and disadvantages; and (3) cooperation conquers competition. The first centers around the present and possible future aspects of *Poké* *mon GO* that promote physical activity. The second focuses on unwanted aspects and specific threats to safety when playing the game. The third shows that cooperation and togetherness are highly valued by the participants and that competition is fun but less important.

**Conclusions:**

Components from *Poké* *mon GO* could enhance the efficacy of physical activity interventions. Cooperation and exploration are aspects of the game that preferably could be transferred into interventions aimed at promoting children’s physical activity.

## Introduction

Participation in physical activities has multiple health benefits for children [1]. It also provides cognitive benefits, including facilitating learning and academic achievement [2,3]. The World Health Organization recommends that children be physically active for at least 60 min daily [1]. Unfortunately, the majority of children around the world do not reach this level [4]. Excessive use of technology has been suggested as a contributing factor to childhood inactivity [5]. However, research also recognizes that games that incentivize exercise have the potential to promote children’s physical activity [6].

*Pokémon GO* is a mobile game where fictional Pokémon creatures are captured and trained [7]. Niantic, the developer, uses an augmented reality technique where live direct views of a real-world environment are augmented by computer-generated graphics to create a geocaching (ie, geographic search) game where Pokémon characters are sought. Supplementing the real world with computer imagery allows players to become immersed in the game, requiring them to be physically active to capture and hatch new Pokémon [7]. Some studies suggest that *Pokémon GO* increases physical activity and discourages sedentary behavior among adults [8-11]. However, another study showed that although the number of steps increased at the beginning, it gradually decreased and returned to previous levels 9 weeks after downloading the game [12]. Nevertheless, the authors conclude that the effect of *Pokémon GO* on physical activity might be different in children [12]. Moreover, the game might have the potential to reach populations that traditionally have low levels of physical activity (preteens and teens) and promote their physical activity [8]. Despite the potential benefits of the game, negative effects, such as increased risk of injury, abduction, and trespassing, threaten the safety and physical well-being of children [13,14]. Further research with children is needed to understand which game components promote initial and sustained physical activity [7]. To the best of our knowledge, this is the first qualitative study of both children and parents aiming to develop an understanding of the underlying mechanisms of *Pokémon GO* so that this knowledge can be transferred to physical activity interventions for children.

We aimed to explore children’s and parents’ experiences playing *Pokémon GO* to determine which components make physical activity games attractive.

## Methods

### Design

This qualitative study is one of the several studies of the process of developing gamification-inspired programs to promote physical activity in children. Qualitative methods provide the opportunity to achieve rich descriptions, enhance understanding of a phenomenon, and capture the voices of people who are rarely heard (ie, the children) [15]. Thus, a qualitative approach to gamification research has the potential to identify the components that make physical activity games attractive to promote physical activity among children and adolescents.

### Participants

Children aged 7 to 12 years and their parents living in a municipality of approximately 80,000 inhabitants situated in north Sweden with experience playing *Poké* *mon GO* were selected using purposeful sampling. Eight families comprising 13 children and 9 parents agreed to participate. Participant information is shown in Table 1. Krueger and Casey [16] recommended having a homogeneous composition of focus groups in terms of age and sex, for example. However, we chose to distribute participants in the focus groups based on family affiliation because we wanted to capture the reflections from the parents on the opinions of the children and vice versa. We paid extra attention to letting the children tell their stories because they could automatically have a sense of less power in this situation.

### Data Collection

In December 2016, data were collected using focus groups. A semi structured interview guide was constructed to ensure all aspects of the aim were addressed. The opening question was, “Let’s pretend I know nothing. Could you please tell me about *Poké* *mon GO*?” To persuade informants to expound on their answers, follow-up questions, such as “Could you tell me more?” and “How did you experience that?” were asked [17]. Each family formed a focus group consisting of 1 or 2 children and 1 or 2 parents, and the interviews lasted between 30 and 60 min. The focus group interviews were performed by the first and last authors, and they were audio recorded and transcribed verbatim.

### Data Analysis

The qualitative latent content analysis was inspired by Graneheim and Lundman [18]. First, transcriptions were read several times to obtain a sense of the overall data; second, the text was divided into meaning units; and third, the meaning units were coded, and these codes were compared, contrasted, and sorted into themes while maintaining fidelity with the text. To strengthen the credibility of the study, the first and last authors initially performed their analyses separately. Then, differences in coding and sorting were discussed, and consensus was reached. The second and third authors made comments about the result to add to readability and clarity. To further strengthen the credibility of the study, quotes were used to illustrate our interpretations.

### Ethical Approval

This study was performed in accordance with the principles of the World Medical Association’s Declaration of Helsinki: Ethical Principles for Medical Research Involving Human Subjects. Each participant signed an informed consent before participating in the study. This study was approved by the Regional Ethical Board in Umeå, Sweden, before the start of the research project (issue date: February 9, 2016; Application Registration Number: 2015/296-31Ö).

**Table 1:** Characteristics of the children and parents who participated in the focus groups.

Numbers of informants	Age in years, mean (range)	Education	*Pokémon GO* experience	
			Time played in months, mean (range)	Level, mean (range)
Boys: 9	9.1 (7-12)	First to sixth grade	3.1 (2-4)	22.7 (17-29)
Girls: 4	9.8 (9-11)	Third to fifth grade	3.2 (3-4)	12.0 (4-22)
Parents: 7 mothers and 2 fathers	38.7 (31-49)	Higher education (7) or high school (2)	2.9 (1-4)	17.4 (0-30)

## Results

The analysis rendered the following three themes: exciting and enjoyable exploration, dangers and disadvantages, and cooperation conquers competition.

### Exciting and Enjoyable Exploration

As the game requires exploration of the participant’s neighborhood for fictional characters that appear and “hatching” of Pokémon eggs, almost all respondents described the game as increasing their physical activity, especially when the game was new. In the beginning, it was often played every day, and sometimes weekend trips were taken with the sole goal of catching Pokémon. It was fun and exciting to catch a new Pokémon and add it to the Pokédex. Having a full Pokédex was a goal for some players; however, with the release of new Pokémon, this was difficult. Having captured a Pokémon that nobody else had gave feelings of uniqueness and gave extra spice to the game. In addition, it was exciting to evolve a Pokémon by giving it candies, which players earned by being physically active. Some players said they walked hundreds of kilometers on “Poké-walks” to catch Pokémon, get candies, or incubate eggs, not knowing which Pokémon would be gained, as shown in the quoted text below:

Boy: The game is really exciting. A couple of days ago, we saw that Pikachu was nearby, and we hurried up with clothes and ran there. But he had disappeared when we came there, and we got really disappointed. Suddenly, he appeared again and I just shouted out. A woman was walking by and just looked at me, but I was so involved in the game.

Focus Group 6

One highlighted advantage of *Poké* *mon GO* was that it combines playing with being outside and moving around. Parents often encouraged their children to play and vice versa. The children wanted their parents to come along on Pokémon walks. Parents told how easy it was to get their children to go for a walk or join a trip to the city, which earlier had not been so appreciated. Some parents, primarily those who played together with their children, began to take extra walks during lunch breaks or in the evenings just to progress in the game. In that way, the game led to increased everyday physical activity, as shown in the quoted text below:

Father: We went on a walk last night, all three of us. I think we were outside one hour. Earlier, we have never gone walking that far and especially my son has not earlier been the one pushing us to go out walking.

Focus Group 4

Both children and parents stated that to keep their interest, it was important that new things happen in the game. Examples were the ability to catch rare new Pokémon or participate in special events that appeared in the game. For instance, on Halloween, more experience points (XP) and candies could be earned. Moreover, bonuses for playing every day during a certain period increased the desire to play. A common wish for future game development was allowing friends to trade Pokémon. As some Pokémon exist only on one continent, it was desirable to have a chat function that allowed people from various parts of the world to connect and exchange Pokémon. In addition, players expressed the desire to collaborate with others and play together, build a joint Pokédex, and so on. Furthermore, they suggested having a map so that friends could be located. Another wish for development was having the ability to do more with their Pokémon than fight in a gym, as shown in the quoted text below:

Father: I think it would be interesting if you could trade Pokémons with each other. The game started as a trading game, and you could trade cards with each other and collect them.

Mother: What if you could get to know someone in, for example, USA, and they could catch one for you there, that doesn´t exist here? Or if a family or a group of friends could play in the same team and have a joint Pokédex.

Girl: It would have been so much more fun if you were able to play together with friends. Mother: Exactly, and I think that this could be motivating for physical activity.

Focus Group 1

### Dangers and Disadvantages

A few children described having small accidents, such as falling into a ditch or biking into a mailbox, when playing. Furthermore, some parents were afraid that their children would walk into a road with traffic without acknowledging the consequences. Some parents limited where their children could play the game, and one parent did not allow his son to go outside alone in the evenings when it was dark. When playing the game, it was difficult to heed one’s surroundings, making it possible to hurt oneself. In addition, one could miss things in nature, such as a squirrel or something else in the real world. One family acknowledged that walking around with a phone in hand at all times increased the risk of theft. Some parents noticed that by playing the game, it became acceptable to walk around with the phone in one hand even when not playing the game, as shown in the quoted text below:

Mother: I think it is bad that the game is built on having the phone in the hand when moving around since it makes it ok for the children to have the phone in the hand in other situations too, like when playing Clash of Clans or using Snapchat. Girl: Yes, but Snap is social. Mother: I know, but earlier you sat down, and walking around with the phone makes you not pay attention to your surroundings. For example, my son can go downstairs with his eyes on the phone, and one day he could break his neck falling down, and Pokémon GO has opened up this behavior.

Focus Group 3

Overall, parents emphasized the challenge in limiting the time their children spent playing computer and mobile games. As they saw several advantages of being physically active and being outside, they were less likely to limit the time spent on this game. In addition, parents who played with their children relaxed their rules about screen time. Moreover, some families experienced conflicts when playing the game. For instance, one boy became hysterical when he was disallowed from going outside to catch a rare Pokémon that he really wanted, as shown in the quoted text below:

Mother: My sister sent a message that a really rare Pokémon was in the city, and my son really wanted to go there, and we couldn´t. It ended in a real fight, and he got really sad, which I can understand because he really wanted that. I have also heard from friends about children becoming hysteric, as it is important to them to catch a Pokémon somewhere. It is like an obsessed behavior.

Focus Group 5

Some families mentioned that others cheat in the game. Two boys gave up playing when it became obvious that some of their friends were cheating, and they thought, “why bother playing” when it is possible to progress in the game incorrectly. Another family refrained from using cheats because they took pride in making progress without using them. A practical problem that occurred with many parents was that their children stayed out playing until the phone battery died. When this happened, the parent and the child could not contact each other in the usual way. This was a particularly large problem in the winter when phone batteries are much less effective. It also led to a need for keeping the phone in a pocket, making it more difficult to play. In addition, several respondents commented on the relative ease of playing in the summer when it was more comfortable because less clothing was needed to play outside, especially for a long time.

### Cooperation Conquers Competition

Cooperation and togetherness were highly valued by the participants, and without a doubt, this was highlighted before competition. Nevertheless, winning a gym fight and competing against other players were mentioned as fun activities, especially among the boys in the study. Through these features of the game, they could measure who was best in the area. Those players who liked competing in the gym were motivated to reach higher levels in the game. One aspect of the game that was especially appreciated was that it led to new friends. The children talked a lot about the game while at school, discussing things such as who found or scouted a rare Pokémon. This brought about new friendships throughout the school. Showing others a rare Pokémon was described as fun, and others were interested in hearing about it. Sometimes, lot of attention was given by people they did not know. At some locations, many players would gather, and it was common for players to start talking to others in the same manner that dog owners start talking to each other about their dogs. For one boy, this game facilitated making friends, something that he previously found challenging, as shown in the quoted text below:

Mother: My boy has had difficulties interacting with peers. Most of them like to play football. Now he has found friends with the same interests, and his social skills grow every day.

Focus Group 7

Some parents and children played on the same phone or using the same login, thereby cooperating in progressing through the game. This added another dimension to the game, and they often talked about which Pokémon was caught, or they were motivated to be more active in the game. Both parents and children described how cooperation was an important factor to continue playing the game, and it motivated them to play a lot. In one family, 2 children and their mother played using a single login, and the daughter would not play at all if not doing it together. Furthermore, cooperation created the opportunity for interacting with the children and talking about other things, such as school, while walking together and playing the game; this is not possible with other computer-related activities. In addition, playing together allowed parents to be more involved in the game compared with other computer or mobile games that their children played, as shown in the quoted text below:

Girl: When I come home and have caught something, I say, “Wow, look at what I have caught.” Boy: Helping each other makes it more fun; it is thanks to mother that we have made this progress in the game. Mother: No, it is thanks to you both and the cooperation that we have reached this far.

Focus Group 2

## Discussion

### Principal Findings

This study shows that the components of the game that the participants valued most were predominately linked to exploring and socializing, and only a few players valued fighting in the gym. We found that the social aspect of the game dominated; comments about walking together, cooperating using a single account, making new friends, and sharing a common interest in the game prevailed. This finding is not in line with the findings of Rasche et al [19], who found that social interaction was not affected by playing the game. In their case, the focus was predominantly on adults, and it is reasonable to believe that children as well as families act differently. The second-most prominent classification was that of the explorers who commented about exploring new places to find new Pokémon, being excited about spotting a rare Pokémon, hatching a new Pokémon, and being able to show off their rare and developed Pokémon. Our findings suggest that gaming elements, such as cooperating and exploring, inspire participation in physical activities. These components of the game can be valuable when designing physical activity interventions using gamification. In earlier research, game characteristics shown to promote physical activity were cooperative play, ﬂexibility in activity choices, short-term goals, integration of physical activities with elements of a story or narrative, rewards, and replayability [7]. Moreover, evidence supports that cooperative gaming offers more intrinsic motivation, self-efﬁcacy, enjoyment, and continued participation than solitary play [20]. An increase in motivation is crucial to physical activity interventions because motivation promotes adherence and attendance, two factors necessary for creating lasting behaviors and physiological changes [20].

Our findings show that playing *Pokémon GO* is not without some risk; however, learning to navigate environmental risks is a natural part of growth and maturation. Furthermore, the sustainability of the new behaviors is inherently based on the elements of the game, such as gaining access to new characters or a joint Pokédex. Thus, the increased physical activity evidenced in this study remains largely dependent on the game and is therefore not likely sustainable. The bidirectional and reciprocal nature of the social cognitive theory [21] can explain underlying tenets related to the discoveries in this study. Using the game as the context, participants could begin to model behaviors that lead to success in the game because this experience brings joy to their lives. Players learn from each experience how best to locate, lure, and catch Pokémon. When successful, the behaviors are replicated. Thus, social learning through watching and cooperating with others to catch specific characters helps shape the new behavior of daily participation in physical activities. Visiting new locations and being physically active increased the likelihood of success, and it was the behavior that linked feelings of enjoyment and excitement with the environment even though it was through a virtual, augmented environment. In other words, the game players were rewarded for their behaviors regardless of whether the behavior was modeled by another player or by the game itself. In the presence of self-observation, self-evaluation, self-reaction, and self-efficacy, as features of the game, the interactivity between and among these processes positively influenced motivation and goal achievement [22]. Self-observation, or having an awareness of one’s performance, does not by itself result in a sustainable behavior change, but the inclusion of both regularity and proximity can enhance its effects on motivation [22]. It is the consistency of the success (eg, if I am physically active, then I am more likely to catch a Pokémon) and the timing of the feedback that influences behavior. The components that make physical activity games addictive might be transferable, and gamification components have been used previously to improve the effectiveness of health promotions [23], and promising research demonstrates the use of gamification with the aim of promoting physical activity [24]. For instance, we are currently developing an intervention promoting children’s active school transportation, and that intervention will draw upon the design patterns from *Poké* *mon GO*.

### Limitations

This is a qualitative study that provides a rich description of families’ experiences playing *Poké* *mon GO*, and it therefore enhances the understanding of how mobile games can promote physical activity as well as capture the voices of people who are rarely heard (ie, the children) [15]. This study is limited in that we used only 8 families; however, the last 2 focus group interviews did not add new insights, indicating saturation in the data. This small sample limits the transferability of the results, but the knowledge gained by this study might be transferable to physical activity interventions for children.

### Conclusions

As cooperation and exploration were the motivating aspects of *Poké* *mon GO* that were most appreciated by the families and most inspiring to increase physical activity, they could be advantageous aspects of gamification when building physical activity interventions for children. For example, including elements of surprise and cooperation with friends and parents, such as collecting points or badges together, might be useful. This knowledge is important when designing interventions aimed at increasing physical activity using gamification. However, more research is needed in the area.
